# High-Pressure

**Published:** 1875-09

**Authors:** 


### HIGH PRESSURE.

The most salient characteristic of life in the latter portion of the nineteenth century is its speed, and the question to be considered is—first, whether this rapid rate is good one, and next, whether it is worth the price we pay for it? No doubt we "do" more, but is "doing" everything, and "being" nothing? The first point to notice is, that we have got into a habit of valuing speed as speed, with little reference to the use made of the time gained. Baron Hubner describes how, in spite of frequent exhortations, the steamers across the Atlantic face all the dangers of icebergs met in fogs, instead of steering a more southern course and arriving but forty-eight hours later. The physical consequences of this needless hurry are grave enough; the moral consequences are possibly graver still; though both sets of effects are as yet only in their infancy, and will take a generation or two fully to develop. The rapidity of railway traveling produces a chronic disturbance in the nervous system, and the anxiety to be in time, the hurrying pace, cause a daily wear and tear as well as accelerated action of the heart, which kills or injures thousands. The constitutions which are thus enfeebled and impaired we transmit damaged to our children, who add to and pass on the sad inheritance. Heart disease, too common already, may be expected to be more common still. We are, perhaps, most of us conscious at some time of the need to be quiet and alone, but few of us have estimated adequately the degree in which an atmosphere of excitement, especially when we enter it young and continue in it habitually, is fatal to the higher and deeper life; the subtle poison which it disseminates throughout the whole character; how it saps solidity and strength of mind; how it daily becomes more necessary and in increasing measure; how it enfeebles and renders abnormally sensitive the subtle organization of the brain; and how far, by slow and sure gradations, it carries us on toward a mental and moral condition which may justly be pronounced unsound. But "high pressure" is shown even more in our style of work than in our rate of movement. The world is more exacting in its demands from all laborers, except merely manual ones. Success in professional, public, and commercial life demands more strenuous and exhausting toil, sterner concentration, and a more harsh and rigid sacrifice of the amenities which time offers the easy-going than was formerly the case.

The eminent lawyer, the physician in full practice, the statesman, and the politician who aspires to be a statesman, even the literary workman and eager man of science, are now condemned to an amount and severity of exertion, which forces one after another to break off (or to break down) in mid-career, shattered, paralyzed, reduced to premature inaction or senility. What work does for the learned professions anxiety does for the merchant and the manufacturer. The lawyer must make hay while the sun shines; the physician cannot, in middle life, select among the patients whom he has longed for through the years of youth; while the statesman has to undergo a prolonged pressure to attain what Macaulay calls "that closely-watched slavery which is dignified with the name of power." Men who have given up their entire being to this business-labor often lose all capability of a better life, all relish for recreation or contemplation, all true appreciation of leisure when it comes at last, for the faculties of enjoyment, like all others, are apt to grow atrophied with disease. The successful man, too, often with much to retire upon, has nothing to retire to; for literature, science, domestic ties, public and philanthropic interests, nature itself, have been lost sight of during the mad struggle, and these are treasures the key to which soon grows rusty. This ceaselessness and severity of toil gives the prizes of life to men of exceptional physique. Physical and cerebral toughness is the prime requisite. The moderately endowed in brains, in health and strength, are "nowhere;" the slow moving bid fair to be elbowed out of their careers; while the prospect before the dull and the dunces, who are seldom the minority, is growing deplorable indeed. Moderate faculties and moderate health are in the same case. Less than a generation ago families could live with all the comforts and essential elegancies of life on $1000 or $1500, who strive in vain to do so now. The same is true of England, which is a paradise for the great proprietor, the successful merchant or engineer, the popular author, and sometimes for the skillful and energetic journalist; scarcely for the quiet, unaspiring, unpushing, who would fain run a peaceful and contented course. It is easier to make much than to live upon a little; and the contented natures who desire to pass their life neither in making money nor in spending it, who wish to use existence wisely and enjoy it worthily, are in danger of being crushed out of existence between the upper and nether millstone of the well-paid laborers and the lavish expenditure of the noble and ignoble opulent. These people used to be a valuable element in the national life, and there is but one way to preserve them. There would seem to be small hope of obtaining a standard of life truly dignified and worthy, except through such a regeneration in the tastes and sentiments of the opulent and noble, the leaders of fashion, the acknowledged chiefs of society, as should cause simplicity to become "good style," and luxury beyond a certain point, and ostentation at any point to be voted vulgar. The seeds of this moral revulsion are already in existence. A few bright and resolute examples among the universally admired might make them germinate with a rapidity that would amaze us.

